# New strategies for *Leptospira* vaccine development based on LPS removal

**DOI:** 10.1371/journal.pone.0230460

**Published:** 2020-03-27

**Authors:** Fabiana Lauretti-Ferreira, Paloma L. D. Silva, Naiara M. Alcântara, Bruna F. Silva, Isabele Grabher, Gisele O. Souza, Erika Nakajima, Milena A. Akamatsu, Silvio A. Vasconcellos, Patricia A. E. Abreu, Eneas Carvalho, Elizabeth A. L. Martins, Paulo L. Ho, Josefa B. da Silva

**Affiliations:** 1 Bioindustrial Division, Butantan Institute, São Paulo, Brazil; 2 Laboratory of Bacteriology, Butantan Institute, São Paulo, Brazil; 3 Laboratory of Bacterial Zoonosis, School of Veterinary Medicine and Animal Science, University of São Paulo, São Paulo, Brazil; 4 Laboratory of Process Development, Butantan Institute, São Paulo, Brazil; UFPL, BRAZIL

## Abstract

Pathogenic spirochetes from genus *Leptospira* are etiologic agents of leptospirosis. Cellular vaccines against *Leptospira* infection often elicit mainly response against the LPS antigen of the serovars present in the formulation. There is no suitable protein candidate capable of replacing whole-cell vaccines, thus requiring new approaches on vaccine development to improve leptospirosis prevention. Our goal was to develop a whole-cell vaccine sorovar-independent based on LPS removal and conservation of protein antigens exposure, to evaluate the protective capacity of monovalent or bivalent vaccines against homologous and heterologous virulent *Leptospira* in hamster. *Leptospire* were subjected to heat inactivation, or to LPS extraction with butanol and in some cases further inactivation with formaldehyde. Hamsters were immunized and challenged with homologous or heterologous virulent serovars, blood and organs were collected from the survivors for bacterial quantification, chemokine evaluation, and analysis of sera antibody reactivity and cross-reactivity by Western blot. Immunization with either heated or low LPS vaccines with serovar Copenhageni or Canicola resulted in 100% protection of the animals challenged with homologous virulent bacteria. Notably, different from the whole-cell vaccine, the low LPS vaccines produced with serovar Canicola provided only partial protection in heterologous challenge with the virulent Copenhageni serovar. Immunization with bivalent formulation results in 100% protection of immunized animals challenged with virulent serovar Canicola. All vaccines produced were able to eliminate bacteria from the kidney of challenged animals. All the vaccines raised antibodies capable to recognize antigens of serovars not present in the vaccine formulation. Transcripts of IFNγ, CXCL16, CCL5, CXCL10, CXCR6, and CCR5, increased in all immunized animals. *Conclusion*: Our results showed that bivalent vaccines with reduced LPS may be an interesting strategy for protection against heterologous virulent serovars. Besides the desirable multivalent protection, the low LPS vaccines are specially promising due to the expected lower reatogenicity.

## Introduction

Pathogenic species of the genus *Leptospira* are the etiologic agents of leptospirosis, an emerging zoonotic disease affecting humans and animals in a global scale. Reservoir hosts carry the pathogens in their renal tubules and excrete them in the urine intermittently, allowing infection of new hosts by the contact of abraded skin and mucous membrane directly with infected urine or indirectly with contaminated water or moist soil, since leptospires show outdoors persistence [1]. As accidental hosts, humans can be infected with *Leptospira* resulting from asymptomatic manifestations to severe and life-threatening conditions such as leptospirosis-associated pulmonary hemorrhagic syndrome (LPHS) and Weil’s disease, involving jaundice, renal failure, and hemorrhage [2–4].

Human leptospirosis is endemic in tropical regions and outbreaks are often associated with inadequate home and sanitation conditions, besides extensive flood events after rainfalls contributing to bacterial dissemination, exacerbating the risk of exposure to urban and rural communities. It is estimated that more than one million cases of leptospirosis occur annually, suggesting that the disease is among the most important zoonosis, causing human morbidity with expressive mortality levels. However, the majority of zoonotic diseases are underestimated due to misdiagnosis and occurrence in poor resource regions [5–7].

As Gram-negative bacteria, *Leptospira* present a cell surface composed of an outer membrane containing lipopolysaccharides (LPS), functional and structural proteins [8]. The carbohydrate moiety of leptospiral LPS is the basis for serological classification of more than 300 serovars of *Leptospira* spp., defined by cross-agglutinin absorption test (CAAT). Human and mouse members of Toll-like receptors (TLR) families have been shown to have distinct ligand specificities for molecular structures such as lipopeptides (TLR2), and LPS (TLR4) [9–10]. The importance of several TLRs for immune response to microbial molecules in vitro have been demonstrated, but their role on the *Leptospira* infection in vivo seems to be more complex [4, 11].

Even after a century of research, only killed whole-cell vaccines (bacterin) are currently worldwide licensed for animal use, such as cattle, swine and dogs [12]. The use of bacterin vaccines for humans is available in some regions (China, Japan, Cuba and Europe), where immunization is restricted to individuals at high occupational risk or in local epidemics events due to its reatogenicity. Even so, annual vaccinations are recommended in all cases, since these vaccines are serovar-specific and induce short-lasting immunity due to the T-independent nature of the response elicited by LPS [3,13]. An approach to overcome this drawback is to reduce the endotoxicity of whole cell vaccines by extraction of LPS.

The availability of complete genome sequences of various pathogenic *Leptospira* species allowed the studies of recombinant proteins as vaccines reveling some promising antigens [14–15]. Initially, the studies focused on the most abundant outer membranes proteins, such as LipL32, OmpL1 and LipL41. They have failed to protect against homologous or heterologous *Leptospira* or contribute to leptospiral clearance in experimental animal challenged [16–18].

The leptospiral immunoglobulin-like proteins, LigA and LigB, have been extensively studied in recombinant vaccines due to their protective immune response, but they have a limited cross-protection considering all pathogenic *Leptospira* spp [14]. In addition to the abundant antigens, a wide range of leptospiral proteins with unknown function have been target of reverse vaccinology studies [2,13,17,19].

Considering that leptospiral surface present numerous antigens and that the main immune response during infection is against LPS, our main goal is to develop a low LPS whole-cell anti leptospirosis vaccine to stimulate the immune response to the conserved protein antigens among different serovars, thus promoting serovar independent cross-protective immunity.

## Materials and methods

### Maintenance of *Leptospira* strains

The *L*. *interrogans* sv Copenhageni (ATCC^®^ BAA-1198^™^) and *L*. *interrogans* sv Canicola (strain LO4, obtained from the Laboratory of Bacterial Zoonosis, School of Veterinary Medicine and Animal Science, University of São Paulo, Brazil), used in this study were successively inoculated in golden hamster (*Mesocricetus auratus*) in order to maintain their virulence, as previously described [20]. Briefly, leptospires were isolated from infected animals and cultured in liquid Ellinghausen-McCullough-Johnson-Harris (EMJH) medium [21], under aerobic conditions at 30°C. After 5–6 days of culture growth, bacteria concentration was determined by cell counting in Petroff-Hausser chamber and suspensions were used for experimental infections.

### LPS removal treatment and vaccine preparation

For preparation of *L*. *interrogans* serovars Copenhageni and Canicola vaccines, one liter of culture were prepared in four bottles by adding 25 mL of five days culture of a serovar into 250 mL of EMJH medium in 1 L bottles for growth at 30°C. After seven days of growth, cultures were analyzed under a dark field microscope to verify viability and absence of contaminants. The number of *Leptospira* was counted in a Petroff-Hausser chamber, cultures were centrifuged at 6,000 rpm for 15min, and pellets of cell were washed sequentially three times with sterile PBS. Cell suspensions were then distributed in equal volumes and submitted to the following treatments: a sample was inactivated by heat at 56°C for 30 min (Heat), other sample was treated with Butanol 8% (But), and a third sample was treated with Butanol 8% and inactivated with Formaldehyde 1% (ButForm). Aliquots of samples from the different treatments were tested for bacterial viability by seeding in EMJH medium and observed for 45 days. Additionally, butanol-treated samples were compared to heat-inactivated and untreated samples to evaluate LPS content measured by purpald assay [22]. All three treatments were applied to individual Can or Cop or combined CanCop serovars, resulting in nine types of vaccines were prepared with one or the two serovars, Copenhageni and Canicola, with same treatments, designated as: CanHeat, CopHeat, CanCopHeat, CanBut, CopBut, CanCopBut, and CanButForm, CopButForm, CanCopButForm.

### Experimental immunizations of hamsters and challenges

Experimental protocols were approved by the Ethical Committee on Animal Use of Butantan Institute (CEUAIB), authorization certificate number 1314/14. This study was performed according to the guidelines outlined by the Brazilian National Council for Control of Animal Experimentation (CONCEA). The approved protocols describe the euthanasia method of the animals by controlled flow rate of CO_2_ in closed chamber. Animals were manipulated and daily monitored by trained personnel. The animals were obtained from animal house of Instituto Butantan.

For immunization and challenge assays, experimental groups of four or five animals were housed per cage inside a ventilated cabinet under controlled temperature and light cycle (12/12 hours, light/dark cycle). Food and water were available without restriction.

All experiments were performed with four experimental groups, according to the cells treatment and vaccine preparations: a non-immunized group (NI), a group immunized with heat-inactivated bacteria (Heat), a group immunized with butanol-treated bacteria alone (But) and a group immunized with butanol-treated bacteria and further inactivated by formaldehyde (ButForm). Doses of vaccine were formulated with approximately 10^8^ *Leptospira* per dose, considering the initial count. The animals were observed and weighed during the experiments to verify possible adverse effects of the vaccines.

Following the detailed outline of strains used as vaccines and challenges (Table 2), hamsters 25 days old were immunized by two doses within 10 days interval. Approximately 300 μL of blood were collected by phlebotomy of the retro-orbital venous plexus before each dose of immunization, and 15 days after the second dose, just before challenge with the virulent *Leptospira*. In some assays the same strain used for immunization was used for challenge (homologous) and in others different strains were used for immunization or challenge (heterologous).

Non immunized animals usually died around 5 to 10 days after the infection. Thirty days post-challenge survivor animals were euthanized and approximately 1 mL of blood was recovered by heart puncture. Kidneys were collected in plastic tube and immediately submerse into liquid nitrogen, and kept to -80 °C for further analysis.

### RNA manipulation and cDNA synthesis for cytokine analysis and leptospire detection in kidneys, by real-time PCR (qPCR)

Samples of the kidneys collected from the hamsters were frozen immediately in liquid nitrogen and stored at -80°C for RNA extraction. For quantification of *Leptospira* in the kidneys of the surviving hamsters, total DNA was purified using DNA extraction kit DNeasy tissue (QIAGEN). Purified DNA from *L*. *interrogans* were used to construct a standard curve. Triplicates of 10-fold serial dilutions (10^−1^ to 10^−8^) of 100 pg initial concentration of genomic DNA from *L*. *interrogans* sv Copenhageni or sv Canicola, contained 3.7 × 10^8^ leptospires were used to construct a standard curve for determination of leptospires DNA in the kidney. The last dilution that could amplify the target at threshold cycle (Ct) was selected as the Lower Limit of Detection. Data of Ct value at different concentrations of DNA were submitted to regression analyses and the equation was used to calculate the leptospires in samples of kidney from hamsters submitted to different treatments.

Total RNA from kidney samples was isolated using Trizol reagent (Invitrogen), and aliquots of RNA underwent DNA digestion treatment using DNAse enzyme (Fermentas) at 37°C for 30 min. As control, RNA was also purified from *Leptospira* cultured 5 days in EMJH at 30°C. An aliquot of 1 μg RNA of each sample was retrotranscribed to cDNA using SuperScript III (Invitrogen) for detection and expression of specific chemokines. Aliquots of cDNA were stored at -80°C until the quantification by quantitative PCR (qPCR). The qPCR reactions were carried out with Syber Green Master Mix (Applied Biosystems, USA), using cDNA diluted to 1:10, and 4 μmol of each forward and reverse primers in 12 μL final reaction. The specific oligonucleotide sequences are presented in Table 1. The qPCR analysis was performed using the Applied Biosystems 7300 Real-Time PCR System. The thermal cycler conditions and melting curve analyses were performed as previously described [23–24]. Consisted in a initial cycle of 50°C/2min, followed by one cycle of 95°C/10 min and 40 cycles of 95°C/15 seconds, 60°C/1 min and 72°C/1 min, and one final step of 95°C/15 seconds, 60 °C/20 seconds and 95°C/15 seconds, 60°C/15 seconds for melting curve analyses. Cycle threshold (C_T_) values for specific genes were normalized to the C_T_ values of chemokine genes expression with *gapdh*. Raw fluorescence PCR data were exported after analysis and PCR efficiency was determined in each individual reaction, using LinRegPCR software [25]. All oligonucleotides had the correlation coefficient squared (R^2^) superior or equal to 0.998 and the range of the efficiency was 1.8–2.0. Relative levels of mRNA from each selected gene were analyzed using the 2^−ΔΔC^_T_ method described [26]. Real-time PCRs reactions were performed in triplicate.

**Table 1:** Oligonucleotide sequences of specific genes of hamster and ribosomal gene of *Leptospira*.

Gene	foward sequence	Tm °C	reverse sequence	Tm °C	amplicon length
cxcl16	AGCAGCAAGAGGACAAAGAG	62	GAAGGAAGACAATGACCAGGAG	62	99
cxcr6	ATGAGGACTACGAGCCAGAT	62	GTACATGCAGGGCAGAAAGA	62	110
ifn-γ	GCCAGATCGTCTCCTTCTACT	63	GTCTGCCTTGATGGTGTCTATG	63	89
ccl5	GTTTGGGAGCAACAACAACAA	62	TGTGAGGGCCTAAGGTATGA	62	102
ccr5	GTGGAAGCACCTAGACAGATTT	62	TGATCTCTCACCCTGACCTTAT	62	100
cxcl10	GGCCTATGGCTACTCCTAATTG	62	CCTGGAGAATAGTGACCTGATG	62	102
gapdh	TGGTGCCGAGTATGTTGTG	62	CAGTAGAAGGTGTGGAGATGATG	62	110
16s	TTCAGTTGGGCACTCGTAAG	62	CGTGTGTTGCCCTAGACATAA	62	97

### Western blotting with distinct *Leptospira* serovars

Leptospires were cultured in 10 mL of cultures of *L*. *interrogans* sv. Copenhageni (ATCC^®^ BAA-1198^™^), *L*. *interrogans* sv. Kennewich (Pomona LPF), *L*. *interrogans* sv. Canicola (LO4), *L*. interrogans sv. Hardjo (strain Hardjoprajitino), *L*. *interrogans* sv. Icterohaemorrhagiae (strain M20), *L*. *interrogans sv*. Bataviae, *L*. *biflexa* sv. Patoc; were used for preparation of protein extracts. Cells were centrifuged and washed 3 times with PBS. The precipitates were suspended in 500 μL of sterile PBS with 1 μL of protease inhibitor solution (Protease Inhibitor Cocktail, Sigma-Aldrich). Cells were disrupted mechanically by vigorous shaking (Mini-Beadbeater, Biospec) with 0.1 mm beads. The resulting extracts were transferred to new tubes and protein concentration was determined using Bradford Dye Reagent (Bio-Rad). Aliquots of 50 μg of each *Leptospira* extract and 10 μg of LipL32 recombinant protein were applied for SDS-PAGE in 6–20% polyacrylamide gradient gels (BioRad) and transferred to nitrocellulose membranes, that were stained with Ponceau to check the transference, washed and blocked with 10% nonfat dried milk in PBST buffer (sterile PBS with 0.05% Tween 20) at 4°C, overnight, washed 3 times with PBST buffer and incubated for 2 h with sera from immunized or non-immunized hamsters (1:5,000 diluted in 5% nonfat dried milk in PBST buffer). After washing with PBST buffer, membranes were incubated for 1 h with anti-hamster anti-IgG antibody conjugated to peroxidase (1:5,000 diluted in PBST buffer). Membranes were washed 3 times with PBST buffer, and then treated with detection kit solutions (Amersham ECL Prime Western Blotting Detection Reagent, GE Healthcare Life Sciences) and exposed in image system equipment (Molecular Imaging Systems-Gel Logic 2200). Analysis was performed using the Image Station 4000 MM (Carestream).

### Statistical analysis

The One-way ANOVA and Tukey post-multiple comparison tests were applied to assess P-values and significance of chemokine and receptor expression differences in samples analyzed by qPCR. Fischer and Mantel Cox methods were used for analysis of the significance of vaccine protection against challenge in hamsters. For statistical analysis, values outside one absolute deviation around the median (MAD-median method) were considered outlier and discarded. Statistics and plotting of data were performed using Prism software (GraphPad).

## Results

### LPS removal and vaccines preparation

Initially, *Leptospira* were cultured and vaccines were prepared by treatment for inactivation and LPS extraction. The levels of LPS were evaluated by purpald assay [22], that indicated about 45% LPS reduction in butanol-treated vaccines, when compared to heat-treated and untreated samples.

### Immunization and challenge assays

**Fig 1:**
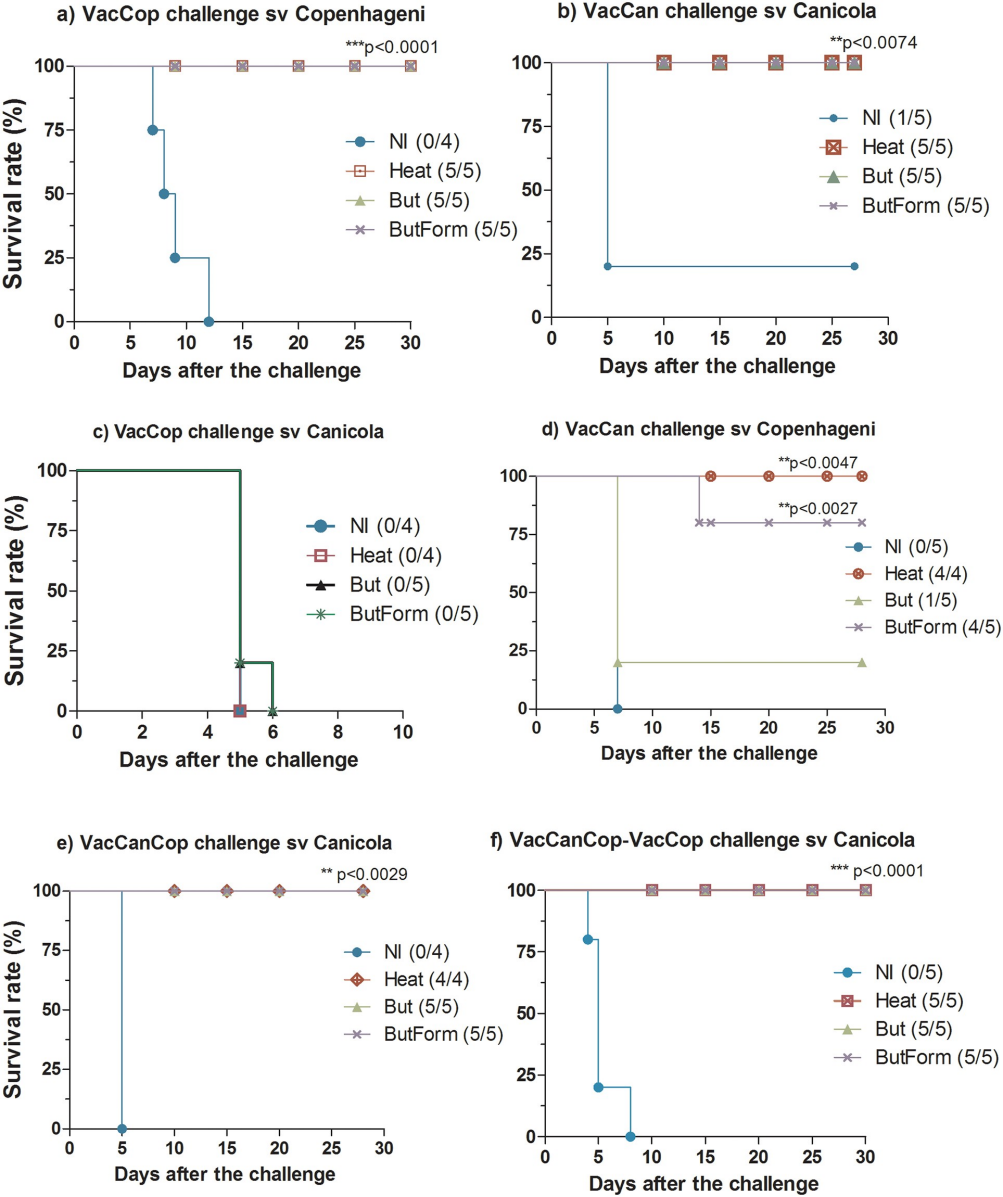
Protection of hamsters immunized and challenged with virulent *Leptospira*; a and b) represent data obtained from animals immunized with *L*. *interrogans* sv Copenhageni and *L*. *interrogans* sv Canicola, respectively, after immunization, these animals were challenged with the virulent serovar homologous to that of the vaccine preparations; c) data from animals immunized with sv Canicola, and challenged with sv Copenhageni (heterologous challenge); d and e) represent data obtained with bivalent vaccines (VacCanCop). The first groups (d) received two doses of bivalent formulations, and (e) received the bivalent vaccine as first dose and second dose contained only the serovar Copenhageni (VacCop). Animals represented in graphic (d) and (e) were challenged with Canicola serovar after immunizations. Statistical analyzes were performed comparing survival curves by Log-rank (Mantel-Cox), followed by Gehan-Breslow-Wilcoxon test using GraphPad Prism software.

Five experiments of immunization and challenge were performed in hamsters, as shown in Table 2. In the first assays (Table 2A and 2B) the animals were immunized with monovalente vaccines followed by homologous challenge with virulent strains. There was 100% protection of all the immunized animals (Table 2). Survival curves and significance statistical are presented in Fig 1a and 1b and S1 Table.

**Table 2:** Animal assays—experimental immunizations and challenge groups.

Experiment	Vaccine		challenge	Survival/total (%)
	First dose	Second dose		
A VacCop	PBS	PBS	Cop	0/4 (0)
	CopHeat	CopHeat	Cop (homologous)	5/5 (100)
	CopBut	CopBut	Cop (homologous)	5/5 (100)
	CopButForm	CopButForm	Cop (homologous)	5/5 (100)
B VacCan	PBS	PBS	Can	1/5 (20)
	CanHeat	CanHeat	Can (homologous)	5/5 (100)
	CanBut	CanBut	Can (homologous)	5/5 (100)
	CanButForm	CanButForm	Can (homologous)	5/5 (100)
C VacCop	PBS	PBS	Can	0/4 (0)
	CopHeat	CopHeat	Can (heterologous)	0/4 (0)
	CopBut	CopBut	Can (heterologous)	0/5 (0)
	CopButForm	CopButForm	Can (heterologous)	0/5 (0)
D VacCan	PBS	PBS	Cop	0/5 (0)
	CanHeat	CanHeat	Cop (heterologous)	4/4 (100)
	CanBut	CanBut	Cop (heterologous)	1/5 (20)
	CanButForm	CanButForm	Cop (heterologous)	4/5 (80)
E bivalent vaccines	PBS	PBS	Can	0/4 (0)
	CanCopHeat	CanCopHeat	Can	4/4 (100)
	CanCopBut	CanCopBut	Can	5/5 (100)
	CanCopButForm	CanCopButForm	Can	5/5 (100)
F Bi / mono valent vaccines	PBS	PBS	Can	0/5 (0)
	CanCopHeat	CopHeat	Can	5/5 (100)
	CanCopBut	CopBut	Can	5/5 (100)
	CanCopButForm	CopButForm	Can	5/5 (100)

Cop-serovar Copenhageni; Can-serovar Canicola; VacCop-Copenhageni vaccine; VacCan-Canicola vaccine; Heat-treated vaccine; But-treated with Butanol; ButForm-treated with butanol and inactivated with formaldehyde.

All animals presented gain of weight after immunization or challenge, except for the non immunized groups after the challenge, as shown in S1 Fig.

The immunization using VacCanHeat (Table 2- exp D and Fig 1d) conferred 100% protection against the heterologous Copenhageni challenge. On the other way around, VacCopHeat did not protect against Canicola (Table 2- expC and Fig 1c). It is not known the specific differences between these strains that could explain these results, but indeed, we observed different pathological condition in the animals infected with the strain Canicola, with disseminated hemorrhage, jaundice and, in general, earlier death, when compared to infection with the strain Copehageni, which presents severe lung hemorrhage. The LPS reduced VacCan conferred only partial protection in heterologous challenge (Fig 1d and S1 Table).

Bivalent vaccine formulations were designed, consisting in a mixture of both sv Copenhageni and sv Canicola. Two doses of the bivalent vaccines were applied (Table 2 - exp. E) or bivalent as prime and monovalente VacCop as boost (Table 2 - exp. F). In both experiments, all animals survived to the challenge with serovar Canicola (Table 2 and Fig 1e and 1f and S1 Table).

### Analysis of the presence of *Leptospira* in the kidneys of hamsters

The presence of *Leptospira* in the kidney of the survivor animals was not detected by growth in EMJH medium neither leptospiral DNA was detected by qPCR, except in one survival animal from VacCanBut group (Table 2- exp. D).

### Profile of antibodies recognition on *Leptospira* protein extracts

**Fig 2:**
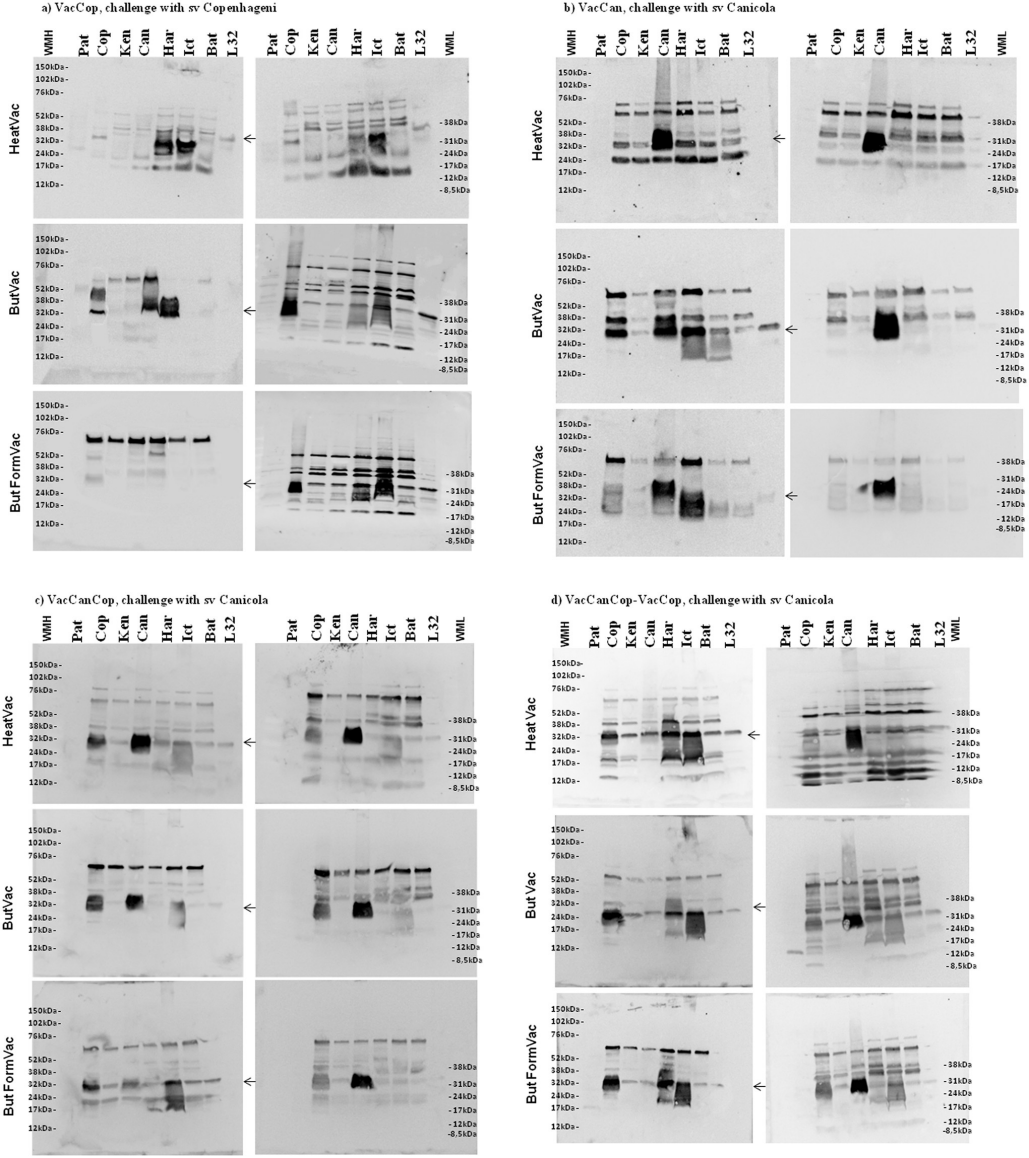
Western blotting of antisera obtained from hamsters immunized and challenged with *L*. *interrogans* serovar Copenhageni or Canicola, analyzed against cell extracts from different *Leptospira* species and serovars. Western blot images were obtained using sera collected from animals of different experimetal groups fifteen days after second dose immunization (left side) and thirty days after challenge (survivor animals) (right side). Immunization and challenge groups as indicated: a) VacCop challenge sv Copenhageni; b) VacCan challenge sv Canicola; c) VacCanCop challenge sv Canicola and d) VacCanCop first dose, VacCop second dose challenge Canicola. Different *Leptospira* cell extracts were prepared, separated by SDS-PAGE in 4–20% gradient gels (except Fig d HeatVac right, that was performed with 12% polyacrylamide). Pat—*L*. *biflexa* sv. Patoc; Cop—*L*. *interrogans* sv. Copenhageni; Ken—*L*. *interrogans* sv. Kennewich (Pomona LPF); Can—*L*. *interrogans* sv. Canicola (LO4); Har—*L*. interrogans sv. Hardjo (strain Hardjoprajitino); Ict—*L*. *interrogans* sv. Icterohaemorrhagiae (strain M20); Bat—*L*. *interrogans sv*. Bataviae (strain Van Tienen); L32—LipL32 recombinant protein; LMW—Low-Range and HMW—Full-Range (ECL Rainbow Molecular Weight Marker (GE Healthcare).

To evaluated cross reactivity of antibodies over proteins of *Leptospira*, the sera collected from hamsters immunized with the VacCan, VacCop or VacCanCop and after the challenge with virulent *L*. *interrogans* sv Canicola or Copenhageni were tested against cellular extracts of different *Leptospira* species and serovars. In general, the antiserum recognized components of all cellular extracts tested (Fig 2a–2d), mainly proteins, with molecular masses approximately 17 kDa, 24 kDa, 38 kDa, 52 kDa and 60 kDa.

### Cytokine and chemokines transcription levels

**Fig 3:**
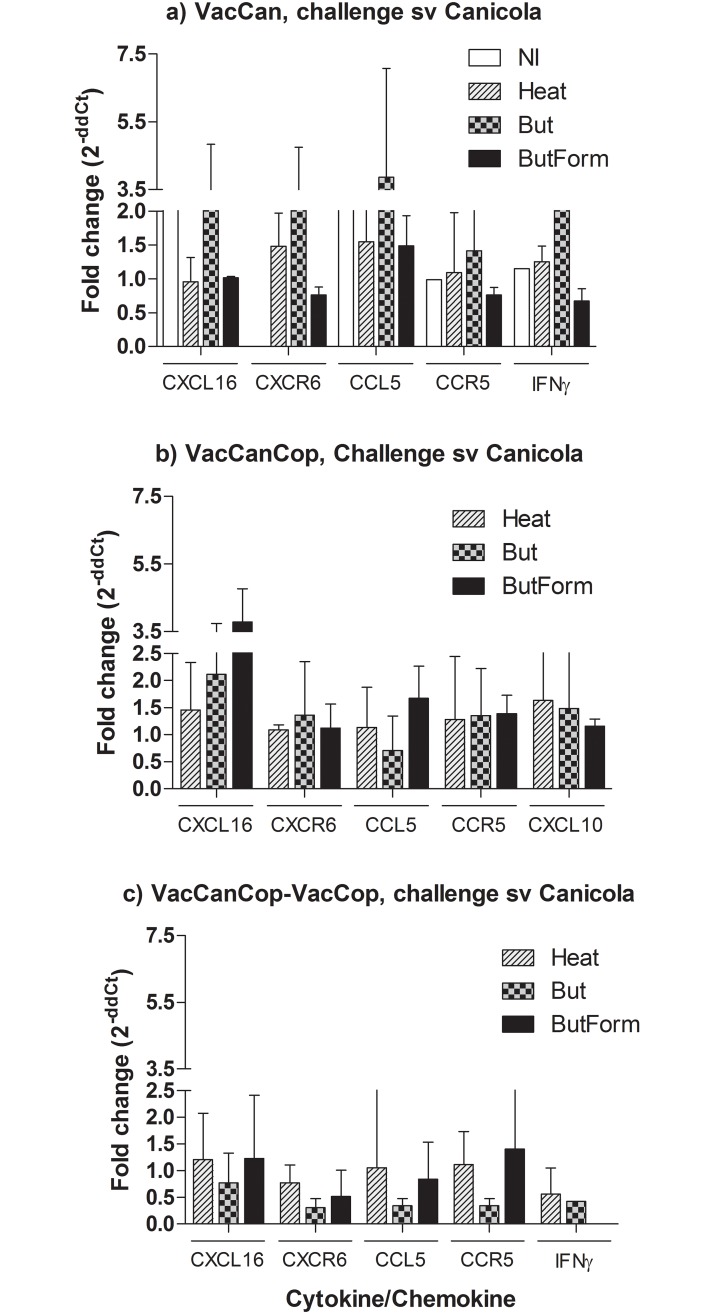
Transcription levels of cytokines, chemokines and their receptors in kidney samples collected from hamsters immunized and challenged with *L*. *interrogans* sv Canicola. Analysis of samples ware performed in triplicates, and two independents experiments. Cycle threshold (C_T_) values for specific genes were normalized to the C_T_ values of chemokine genes with *gapdh* gene.

Transcription levels of the genes coding for cytokines, chemokines and receptors, IFNγ, CXCL16, CCL5, CXCL10, CXCR6, and CCR5, were evaluated by qPCR on samples of kidneys of immunized and challenged hamsters (Fig 3). An increase of the transcripts was observed in all survivors, including the non immunized animal (Fig 3a) that survived to challenge with serovar Canicola challenge, relative to the basal level.

We can highlight a small increase in transcription levels of the CXCL16, CXCR6, CCL5, and IFNγ genes in animals immunized with VacCanBut and homologous challenge (Fig 3a). It was observed an increase in transcription levels of the CXCL16 gene in animals immunized with VacCanCop (Fig 3b and 3c). The levels of the analyzed chemokines were not significantly different in samples of kidney of the animals immunized with the different vaccine preparations.

## Discussion

An important goal on development of leptospirosis vaccines is the induction of cross-protective immunity, and several studies have shown that this can be achieved. Some approaches using live vaccines demonstrated cross-immunity in guinea pigs between the Pomona and Canicola serovars and also between the Pomona and Icterohaemorrhagiae serovars, all belonging to the species *L*. *interrogans* [27]. A similar study showed cross-immunity between several serovars of *L*. *interrogans* and *L*. *kirschneri* species [28]. Later, the same group demonstrated significant but incomplete interserovar immunity among four serovars of *L*. *borgpetersenii* in hamsters.

Similarly, our results immunizing hamsters with heat inactivated serovar Canicola vaccines (HeatVac) or treated with butanol and formaldehyde (ButForm), exhibited at least 80% protection against the heterologous challenge with virulent serovar Copenhageni (Fig 1d). On the other hand, our results showed that immunization with Copenhageni vaccines failed to protect against heterologous challenge with virulent serovar Canicola (Table 2C and Fig 1c). Similar results were described in the literature [29], where cross-protection among unrelated *Leptospira* serovars strain was demonstrated, some cases only partial protection was observed and others cases only homologous protection.

The fact that antisera from immunized animals recognize several proteins in different pathogenic species and serovars of *Leptospira* (Fig 2) supports a cross-immunity data. It is supposed that the antigens targeted for protection during challenge are proteins, since leptospiral lipopolysaccharides are not capable of inducing cross-immunity even among species-related serovars [19, 30].

Other studies using inactivated or fragmented cell-vaccines have observed cross-protective capacity. Sonrier and colleagues showed that whole cell extracts of the serovar Icterohaemorrhagiae were able to induce significant protection against challenge with the Canicola serovar [30] but the LPS fractions did not protect against heterologous serovar. Cross-immunity was described using formalin-inactivated vaccines with serovars Ballum (*L*. *borgpetersenii*) and Canicola (*L*. *interrogans*) induced homologous and heterologous immunity against serovar Copenhageni [29].

Local and systemic reactions of various degrees are reported as side effects of whole cell vaccines due to components of leptospires or reagents present in the culture medium [13, 31]. The reactivity of bacterial vaccines in humans has been a major concern and mandatory use is controversial in many countries. An approach to overcome this drawback is to reduce the endotoxicity of whole cell vaccines by extraction of LPS with butanol. This practice was well-established for *Bordetella pertussis* whole cell vaccines, without affecting the potency, stability and integrity of the product [32], and no significant difference in antibody response was observed when compared to untreated vaccines. Likewise, our butanol-treated vaccines, with or without formaldehyde (But and ButForm), demonstrated the same protective capacity of heat-inactivated vaccines (Heat) in immunization and challenge experiments (Fig 1), including the clearance of leptospires from renal tubules, antibodies production (Fig 2), and stimulation of transcription of cytokines, chemokines and their receptors (Fig 3).

Studies by Srikram and collaborators [33], demonstrated that a live, attenuated vaccine based on a LPS defective *L*. *interrogans* serovar Manilae was able to confer 100% survival of hamsters challenged with non related serovar Pomona, although it did not prevent the renal colonization. The identity of all protective related antigens remains unknown, however, immunization of hamsters with whole cell vaccine raised antibodies response to important membrane proteins such as LipL32, LipL41 and Loa22, which may be the proteins recognized by the sera of immunized animals in our Western blot assays, bands 31 kDa, 38 kDa, and 24 kDa (Fig 2).

Regarding the transcription of cytokine, chemokine and receptor, all immunized and challenged animals present elevated levels of mRNA of evaluated genes in kidney in relation to the control NI, and there was no significant difference among the groups. Stimulation of inflammation is essential for the resolution of microbial infections. Chemokines and their receptors are key pieces to target migration and infiltration of leukocytes and, likewise, proinflammatory cytokines, such as interferon gamma (IFNγ), act as chemoatractant to recruit leukocytes to sites of damaged and/or infected tissue [34]. In fact, chemokines have been used as indicative marker of vaccine inflammation and vaccine immune response [35].

In our study, animals immunized and challenged presented elevated levels of CXCL16 mRNA (Fig 3b). As reported CXCL16 and its CXCR6 receptor play important role in the recruitment of natural killer T cells and in the protection of animals in glomerulonephritis models [23,36], [37]. Studies by Lee and collaborators [38] indicated that expression of CXCR6 on lung T cells after immunization is a marker for local protective immunity to tuberculosis and that this receptor and CXCL16 play an important role at localization of T cells. The CXCR6-cells were antigens specific.

As well reported, CXCL16 is expressed by antigen presenting cells, mostly dendritic cells and macrophages that play several functions involved in the response to bacterial infection. Activated lymphocytes abundantly express CXCL16 and CXCR6 receptor [39]. The CXCR6 expression on CD8^+^ T cell is a critical requirement to establish long-lived memory T-cell population in the liver [40]. Furthermore, cell surface expressed CXCL16 can also act as an adhesion molecule for leukocytes expressing CXCR6 [41]. These molecules support the binding and phagocytosis of Gram-positive and Gram-negative bacteria when expressed by macrophages and dendritic cells [42].

CCL5 is an important chemokine that also increased expression in all analyzed immunized animal. CCL5 and its receptor CCR5 could participate in a positive feedback of the inflammatory response by enhancing the recruitment of macrophages and immature DCs and is critical to start the immune response, and for maintenance of memory response. CCR5 receptor is involved in the recruitment of immune cells as well as non-immune cells under pathological condition [23,43–45].

CXCL10 was detected when the animals were immunized with two doses of bivalent vaccine. Our previous report demonstrated that this chemokine is differentially expressed in mice resistant to virulent *Leptospira* infection.

CXCL10 is important as marker of vaccine elicited inflammation and have been reported as acting in T cell immunity and is critical for the generation of protective CD8 T cell responses induced by activated dendritic cells. It has been shown that the presence of these cells at the site of injection significantly augment antigen specific B and T cell immune responses [35,46–47]. It was reported that CXCL10 is among several inflammatory chemokines induced in vaccinated mice and that it was important for a protective response in Leishmania infection [48].

## Conclusions

Taken together, our results demonstrated that the approach of using bivalent vaccines with strongly virulence serovars of *L*. *interrogans* (Canicola and Copenhageni) associated with reduced leptospiral LPS promotes antibody mediated protection and stimulation of cytokines and chemokines, important for prevention of leptospirosis. It is a promising strategy for development of vaccines that require induction of cross-protective immunity, nevertheless, more in-depth studies are needed to assess the extent of cross-immunity and to identifying the major antigens related to protection. The obtained results contribute to advances in vaccine research and to outline new strategies in the development of vaccines against leptospirosis.

## Supporting information

S1 Fig Weight of hamsters during immunization and challenged with virulent *L*. *interrogans*. (PDF)

S1 Table Significance of vaccine protection: Analyses by Fischer and Mantel Cox methods for significance of vaccine protection against the challenges in hamsters. (XLSX)

## Abbreviations

CCL5: C-C motif chemokine ligand 5
CCR5: C-C motif chemokine receptor 5
CXCL10: C-X-C motif chemokine ligand 10
CXCL16: C-X-C motif chemokine ligand 16
CXCR6: C-X-C motif chemokine receptor 6
IFNγ: interferon gamma
LPS: lipopolysaccharide
