# Supplementary material for: New strategies for Leptospira vaccine development based on LPS removal
Source: PLoS One. 2020 Mar 27;15(3):e0230460. doi: 10.1371/journal.pone.0230460 (PMC7100938; doi:10.1371/journal.pone.0230460)
Supplement: S1 Fig — (PDF) [file pone.0230460.s001.pdf]

Fig. S1

a) VacCop challenge sv Copenhageni

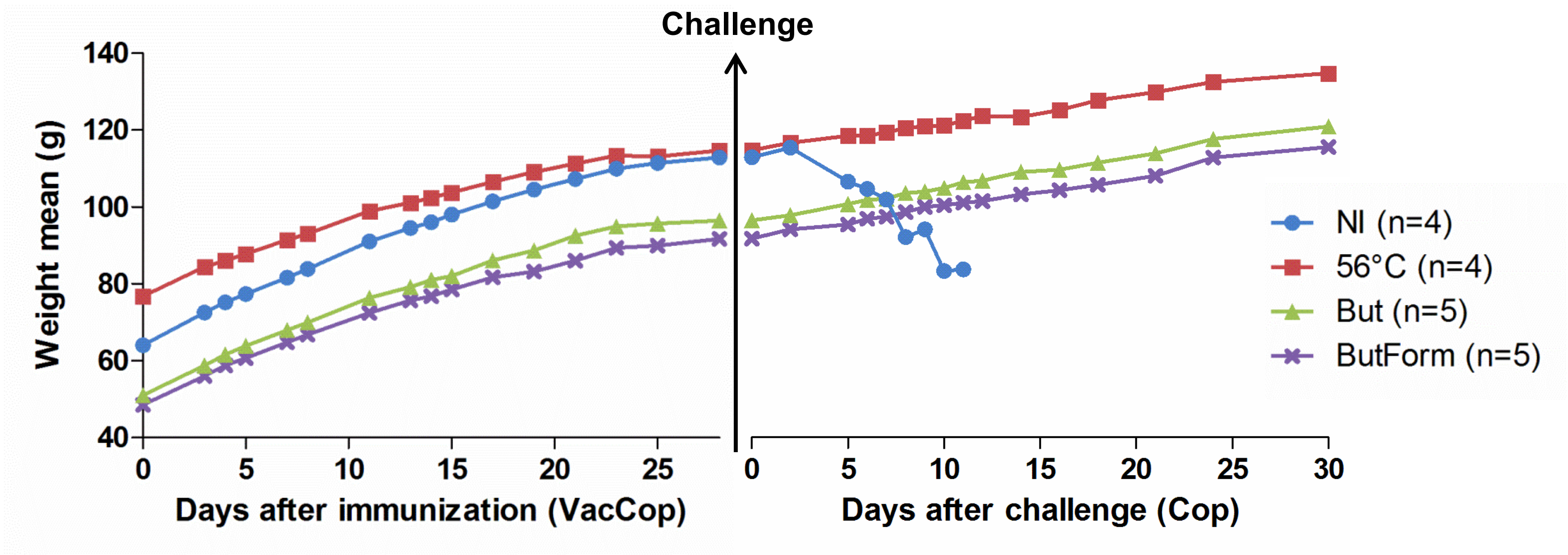

b) VacCan challenge sv Canicola

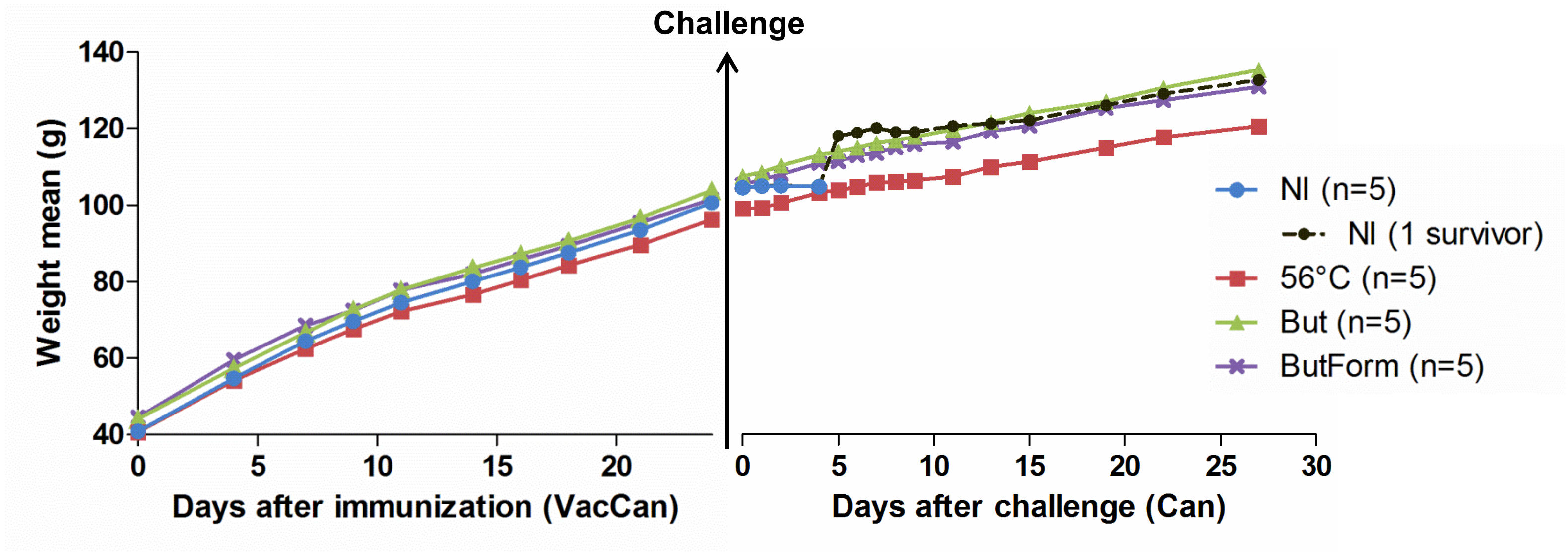

Fig. S1 Continuation

c) VacCan challenge sv Copenhageni

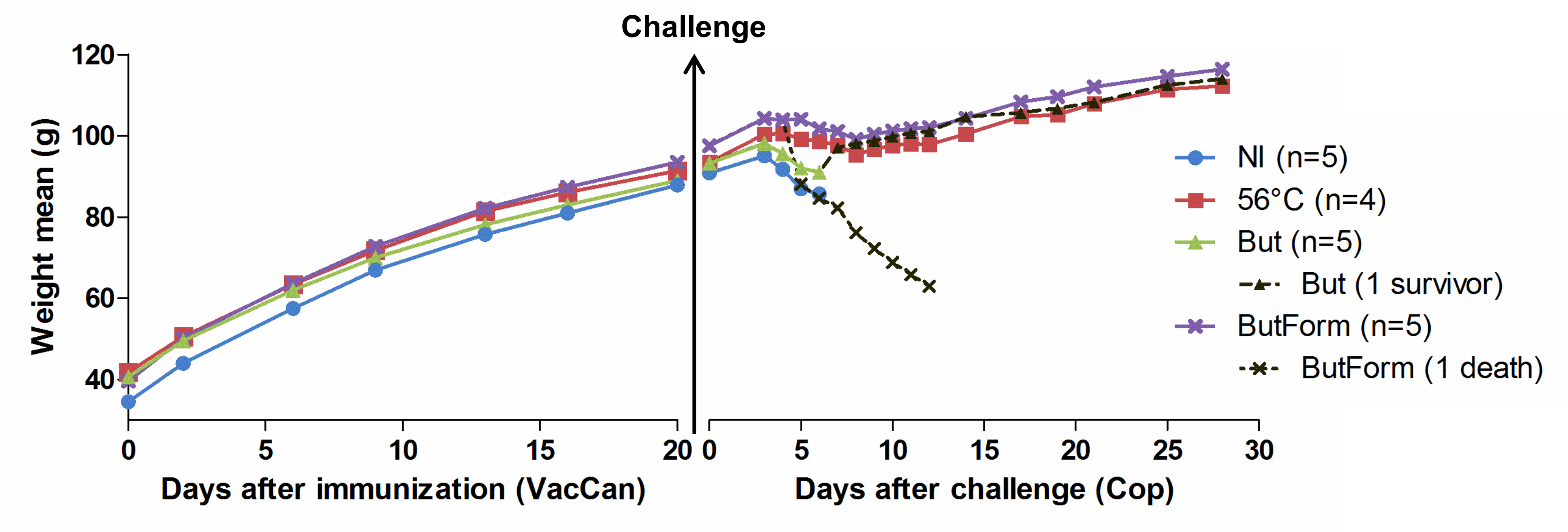

d) VacCanCop challenge sv Canicola

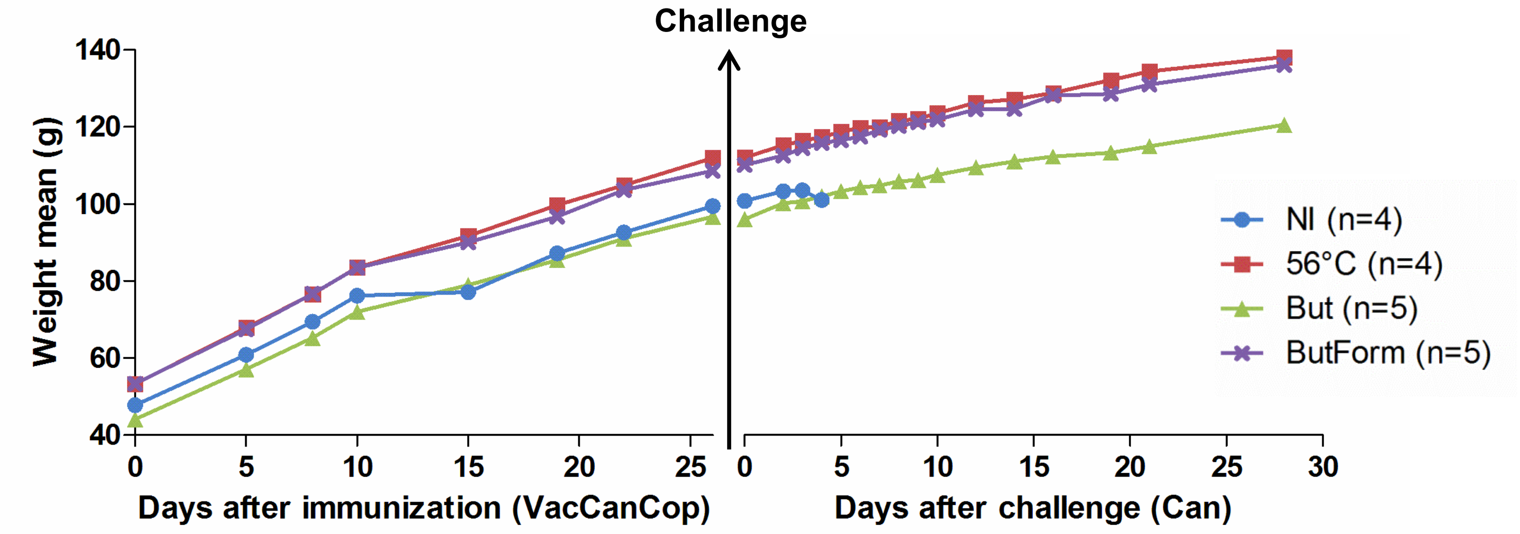

Fig. S1 Continuation

e) VacCanCop-VacCop challenge sv Canicola

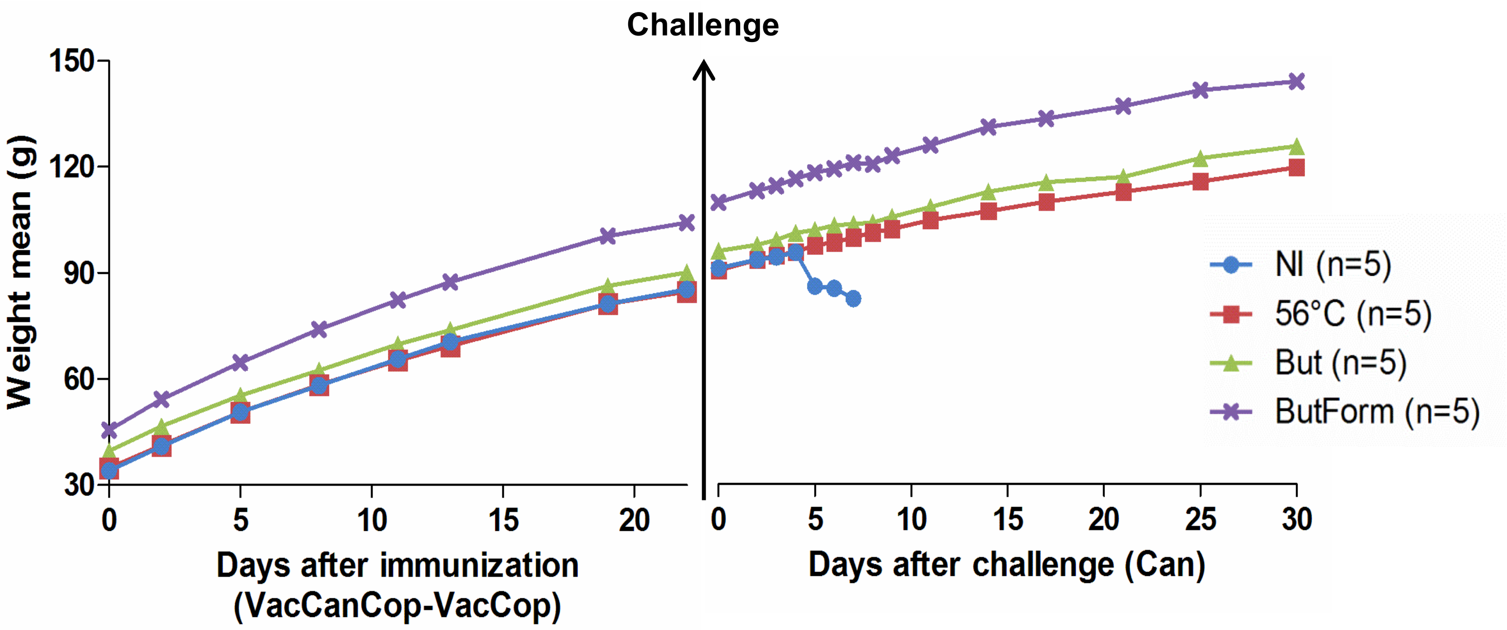

Figure S1. Weight of hamsters during immunization and challenged with virulent *L. interrogans.* a and b) represent data obtained from animals immunized with *L. interrogans* sv Copenhageni and *L. interrogans* sv Canicola, respectively; after immunization, the animals were challenged with the virulent serovar homologous to that of the vaccine preparations; c) Data from animals immunized with sv Canicola, and challenged with sv Copenhageni (heterologous challenge); d and e) Represent data obtained with bivalent vaccines (VacCanCop); The first groups (d) received two doses of bivalent formulations, and (e) received the bivalent vaccine as first dose and second dose contained only the serovar Copenhageni (VacCop). Animals represented in (d) and (e) graphics, were challenged with Canicola serovar after immunizations.
